# The challenge of planning learning opportunities for clinical medicine: a triangulation study in Iran

**DOI:** 10.1186/s12909-019-1719-3

**Published:** 2019-07-31

**Authors:** Tahereh Changiz, Nikoo Yamani, Maria Shaterjalali

**Affiliations:** 1 Department of medical education, Medical educational research center, Isfahan University of Medical Science, Isfahan, Iran; 2 School of Medical Science, Islamic Azad university of Tonekabon branch, Tonekabon, Iran

**Keywords:** Clinical learning opportunities, Student-centered strategy, Clinical teaching-learning methods, Student evaluation, Educational climate

## Abstract

**Background:**

An essential characteristics of clinical education is the need to learn a large number of practical and communication skills along with theoretical knowledge. It is challenging to design learning opportunities (LOs) for clinical setting. We aimed to determine optimal learning opportunities from the viewpoint of Medical curriculum planners, to determine the gap between the current condition and the optimal condition in medical schools, and to present feasible tactic for clinical learning opportunities.

**Methods:**

This study comprised of three sub-studies and was conducted using triangulation. The first sub-study was performed using the Modified Delphi method with a view to identifying optimal learning opportunities. Data was collected by online focus group discussion and a questionnaire. The second sub-study was conducted with the aim of comparing the current condition and the optimal condition. Data was collected from nine medical schools across Iran using a checklist, available documents, observation, and interview. The third sub-study was conducted using an expert panel comprising of seven curriculum planners of the M.D. program. The goal of this phase was to provide feasible tactic to improve clinical education in medical schools.

**Results:**

In the first sub-study, the participants determined all items, including student-centered learning, non-threatening learning environment, and record and management system of clinical learning opportunities as implementable learning opportunities with over 70% consensus. However, in the second sub-study, student-centered teaching methods were practiced in 33% of medical schools and the non-threatening learning environment in 67% of the schools, while the record and management system of learning opportunities was not launched in any of the schools. From the viewpoint of the expert panel members, learning opportunities adapted to clinical contents, specification of content-based learning opportunities, and continuous supervision on learners to achieve the expected learning outcomes were among clinical learning opportunities with over 70% consensus.

**Conclusions:**

Student-centered clinical learning practices, together with virtual learning methods, can lead to clinical enhancement. Opportunities such as interactive and participatory practices should gain further consideration. Also assigning responsibility to learners and monitoring them are strategies for enhancement.

## Background

Clinical education is an essential component of the medical curriculum and a significant part of the health services education [1, 2]. A central feature of the M.D. program is the need to learn a large number of practical and communication skills along with the theoretical knowledge [3]. Learning opportunities (LOs) are provided by clinical educators so that learners can obtain learning outcomes [4]. Nevertheless, to design practical LOs in the clinical settings is extremely challenging. It is because the primary goal in health care centers is to deliver quality care to patients, and LOs are provided on the sidelines for learners who play an auxiliary role in patient care [5]. Nonetheless, as patient care in clinical settings provides an excellent opportunity for learners to acquire clinical, communication, and interdisciplinary skills and to role model after teachers, most medical students often consider clinical education as valuable [6].

The shift from the teacher-centered to the student-centered paradigm and the emphasis on student learning experiences have been among effective strategies to create LOs at clinical settings in recent years. Ernstzen et al. enumerate, from their review of the literature, a number of LOs, including collaborative learning, active participation, responsibility sharing with learners in the clinical environment, informing learners about the process and outcome of learning, problem solving as regards learner tasks, problem solving via actual and hypothetical patients, problem solving via the cyberspace, discussion on patient case, role-playing with peers, case-study assignments, self-directed learning, brainstorming, article reviewing, feedback, self-assessment, and lifelong learning skills [5]. Alweshahi, as quoted by Stritter et al., states that students describe the teaching methods that lead to their better clinical education as a LO. Further opportunities added by Streeter et al. involve cases where the teacher creates a friendly environment in which students are actively engaged [7]. Other studies have also shown that, from the learners’ perspective, clinical learning is associated with issues such as patient management, having a responsibility in the hospital, feeling at work when in a team, working in the environment, and direct exposure to actual patients. Besides, membership in the patient recovery team, having a responsibility, prescription writing, and collaboratory learning are mentioned as fascinating learning experiences [8, 9].

However, challenges such as the limited training time at the patient’s bedside, alterations in the care system, and prioritization of the patient management and his/her treatment can complicate clinical learning and affect medical education as well as a learner’s ability to learn patient care [5]. Moreover, problems such as lack of opportunity to work independently, fewer outpatient visits, greater attention directed to specialized patients, and performance of tasks unrelated to internship responsibilities along with several other ineffectual tasks unrelated to a M.D.’s curricular goals [8] have turned LOs in the clinical setting into a challenge that requires accurate planning and focused attention.

Among the main factors in creating LOs in clinical settings are the design, development, and implementation of opportunities adapted to educational outcomes, their monitoring and evaluation, and the creation of an appropriate educational environment in the clinical setting. However, while there is research on the teaching-learning methods, there is a paucity of research on other dimensions of teaching LOs for the M.D. students. Thus, we were motivated to conduct this research. In this study, we first determine the optimal and feasible LOs in the clinical setting from the viewpoint of the M.D. curriculum planners. Subsequently, while identifying the gap between the current condition and the optimal condition in medical schools, we present strategies for implementing and monitoring LOs and feasible strategies to create them.

## Methods

The present study consisted of three sub-studies and was conducted using triangulation [10, 11]. The M.D. planners in Iran with the positions of vice-chancelleries of education, dean of medical schools, M.D. education deputies, and clerks and interns in all medical schools throughout the country were eligible to participate in this study. The study lasted from May 2016 to June 2018.

### First sub-study

The first sub-study was performed using the Modified Delphi method. The goal of this phase was to provide a list of optimal clinical LOs for students in the M.D. program. The M.D. planners in Iran with the positions mentioned above (vice-chancelleries of education, dean of medical schools, M.D. education deputies) were eligible for this stage of the study, which was conducted in two rounds. The first round was conducted using qualitative content analysis [12] with a directed approach [13, 14]. Participants were selected purposeful with maximum variety. Data collection methods consisted of online focus group discussions (12 individuals) and 8 face-to-face or over-the-phone semi-structured interviews. Online focus groups was performed with two sessions with 6 people in each session. The second round was performed using a 20 statement questionnaire set in two sections. Sampling in the second round was through the census method. At first, electronic questionnaires which were designed by researchers, were sent via e-mail to the participants. To confirm face and content validity, the experts reviewed the statements on several occasions. The number of respondents to electronic questionnaire were 3. Then printed questionnaires were distributed among the participants after a month. The number of respondent were 51. The final number of respondents were 54. The consensus level was to reach a frequency percentage of 70%. Lincoln and Guba’s criteria [15], group statistic, and anonymous responses by participants (in the first round), Alpha coefficients with an emphasis on internal consistency (in the second round), were used to ensure the reliability for each round.

### Second sub-study

The second round was conducted in a descriptive, cross-sectional manner. The purpose of this step was to compare the status quo in clinical education and the optimal condition obtained from the first sub-study results and to enlist and describe the strengths and limitations of clinical education in M.D. programs across the country. The statistical population comprised of M.D. education deputies and clerks and interns across the country. Nine medical schools with different levels were selected by stratified sampling method [16]. The required information on the status quo of LOs in clinical setting was gathered through a data collection form, documents, observation, and informal interviews during four months. One of the researchers conducted the observation. Participants at this stage were M.D. education deputies (9 individuals), clerks, and interns (17 individuals). To enable comparison between the current and the optimal conditions, the forms that collected data on these situations held similar items. The experts reviewed the tool to confirm its face and content validity in several stages. Reliability of the tool was determined in the previous sub-study. To examine the status quo, we analyzed the data collected on the current condition in SPSS (version 20) and the percentage frequency using descriptive statistics.

### Third sub-study

The third sub-study was conducted using the nominal group technique and a panel of experts [17]. The purpose of this phase was to provide practical solutions for improving the clinical education of the M.D. program. The participants of this phase were seven M.D. program planners with a history of education and executive responsibility from the ten national macro-region universities of medical sciences and the representatives from the Secretariat of the General Medical Education Council of the Ministry of Health and Medical Education. In this phase, building on the findings of the first and second sub-studies and a review of the literature, we integrated the idea generation phase and round robin [18–20] and distributed a data collection questionnaire. The questionnaire consisted of two sections including demographic characteristics and items on the strategies to design LOs with three policies and nine tactics on a five-point scale. It was given to the panel members to vote. The questionnaires were repeatedly reviewed and revised by experts to confirm their content and face validity. The data from the questionnaire were analyzed in SPSS (version 20) to determine the frequency and extent of agreement. The results were categorized as implementable in a short-term period (one year or less), implementable in the mid-term period (maximum of five years,) and non-implementable. The reliability of the research was confirmed by selecting individuals from different colleges [18] and collecting comments on anonymous forms and group responses [19].

## Results

Participants of the first sub-study were 20 individuals in the first round and 54 respondents in the second round. The number of code (statements) from the first round was 91 (Table 1). This section of the study has been published [21]. The second round was conducted in two sections: providing optimal LOs with 14 items and the optimal policies for implementing LOs with six items (Table 2). Participants in the second sub-study were nine medical school planners from across the country and 17 clerks and interns from various medical schools. The checklist for comparing the current and optimal conditions had two sections: optimal LOs with 14 items and optimal policies for implementing LOs with six items (Table 3). Participants in the third sub-study were seven medical education experts from across the country. One strategy with three policies and nine tactics was examined in the expert panel and categorized into the three categories of implementable in a short-term period, implementable in the mid-term period, and non-implementable (Table 4).

**Table 1:** Themes, categories, and subcategories of LOs

Main Category	Sub-category	Sub-classes
Creating learning opportunities	Designing and planning LOs	–
	Empowerment and enthusiasm of teachers	–
	Educational atmosphere	–
	Learning strategies	Effective technique
		Clinical reasoning
		Problem-based learning
		Virtual education
		Evidence-based medicine
		Collaborative learning
		Study guide
	Supervision of students	Feedback/Reflection
		Continuous supervision
	Evaluation	Student assessment, Learning recording tools (Logbook/Portfolio)

**Table 2:** Optimal LOs

Learning Opportunities	Efficient and very efficient %
Providing optimal LOs	
Teaching through small group discussion under teacher supervision on actual patients	97.8%
Using clinical reasoning approaches adapted to different clinical setting (e.g., problem-solving)	100%
The teacher discussing the patient case with learners before and after interacting with the patient or performing the surgical procedure(s)	97.8%
Describing clinical problem and illustrating patient management on actual patients	97.8%
Using other student-centered methods (such as role-play, questioning, brainstorming and ….)	95.7%
Using multimedia and educational films for teaching	95.7%
Expressing medical experiences for educational purposes	91.3%
Providing a non-threatening educational environment and building confidence for learners	100%
Encouraging learners to participate actively in the clinical services process to learn more	100%
Applying learning tools tailored to learning objectives and settings (including the study guide and the logbook)	93.4%
Delegating important clinical responsibilities (in line with learning competency and objectives) to learners during learning	95.7%
Continuous supervision on the learner’s performance while she/he is performing clinical tasks	100%
Providing appropriate feedback to learners in clinical settings	97.8%
Assessing interns using a clinical competency assessment	95.6%
Optimal policies for implementing LOs	
Planning for the implementation of LOs as a longitudinal theme in the M.D. curriculum	95.6%
Vertical integration of clinical and basic sciences	78.3%
Launching a record and management system of clinical LOs that is available at all supervisory levels	86.9%
Conducting continuous educational team development on the ways to design and manage LOs	97.9%
Continuously evaluating the educational team by the Teacher Evaluation Unit in terms of how to manage LOs	95.7%
Encouraging members of the educational team to manage LOs effectively	95.7%

**Table 3:** Comparison between the current conditions and the optimal conditions

Optimal conditions	Current conditions (Yes %)
Comparison of the current and optimal situations of LOs	
Teaching through small group discussion under teacher supervision on actual patients	33.3%
Using clinical reasoning approaches adapted to different clinical setting (e.g., problem-solving)	22.2%
The teacher discussing the patient case with learners before and after interacting with the patient or performing the surgical procedure(s)	44.4%
Describing clinical problem and illustrating patient management on actual patients	77.8%
Using other student-centered methods (such as role-play, questioning, brainstorming and ….)	33.3%
Using multimedia and educational films for teaching	22.2%
Expressing medical experiences for educational purposes	88.9%
Providing a non-threatening educational environment and building confidence for learners	66.7%
Encouraging learners to participate actively in the clinical services process to learn more	33.3%
Applying learning tools tailored to learning objectives and settings (including the study guide and the logbook)	33.3%
Delegating important clinical responsibilities (in line with learning competency and objectives) to learners during learning	33.3%
Continuous supervision on the learner’s performance while she/he is performing clinical tasks	66.7%
Providing appropriate feedback to learners in clinical settings	55.6%
Assessing interns using a clinical competency assessment	44.4%
Comparison of the current and optimal conditions of policy-making for the realization of LOs	
Planning for the implementation of LOs as a longitudinal theme in the M.D. curriculum	0
Vertical integration of clinical and basic sciences	0
Launching a record and management system of clinical LOs that is available at all supervisory levels	0
Conducting continuous educational team development on the ways to design and manage LOs	33.3%
Continuously evaluating the educational team by the Teacher Evaluation Unit in terms of how to manage LOs	44.4%
Encouraging members of the educational team to manage LOs effectively	0

**Table 4:** Strategies, policies, and tactics to enhance LOs

Strategy: Designing LOs tailored to each course and based on the expected competency as set in the M.D. program				
Policies and tactics	Short-term (one year)	Mid-term (five years)	No feasible	No idea
Policies				
Adoption of an appropriate educational strategy in the M.D. curriculum to design LOs conforming to clinical contents and learners’ level	42.8%	28.6%	14.3%	14.3%
Provision of continuous educational team development on the design and management of LOs	71.4%	–	14.3%	14.3%
Availability of programs to continuously assess the performance of the educational team and supervision to manage LOs	57.1%	28.6%	–	14.3%
Tactics				
To identify the LOs in line with the contents of the clinical curriculum for clerks and interns	80%	20%	–	–
To implement the vertical integration strategy between clinical science and basic science in the M.D. program	14.3%	42.9%	42.9%	–
To launch a record and management system of clinical LOs that is available at all supervisory levels	50%	16.7%	33.3%	–
To distinguish between educational and therapeutic heath service with an aim to reduce the number of patients in educational health service and teach via collaborative learning and problem-solving	14.3%	42.9%	42.8%	–
To use educational setting with various actual patients corresponding with educational objectives to show ways to manage a patient	28.6%	57.1%	14.3%	–
To use multimedia and educational films to teach how to reduce clinical cases in line with educational goals	85.7%	14.3%	–	–
To use other student-centered educational methods (such as role-play, questioning, brainstorming and …)	28.6%	57.1%	14.3%	–
To provide a non-threatening educational environment and to build confidence for learners	57.1%	14.3%	28.6%	–
To continuously supervise learners to determine expected learning outcomes and to design new educational methods if necessary	57.1%	42.9%	–	–

## Discussion

We did not find many studies on the use of optimal LOs in medical clinical settings. In this research, we both identified the optimal LOs and determined the feasibility of these opportunities. In this section, we discuss the optimal clinical education conditions obtained from the first sub-study in comparison with the current conditions derived from the second sub-study and the solutions derived from the results of the third sub-study in an integrative manner.

### Varying LOs

Our participants specified 14 LOs as optimal, which should be provided to learners in different learning environments. Training via small group discussion on actual patients was an optimal LO. However, this tactic was used only in one-third of the schools. It seems necessary to empower teachers regarding student-centered educational strategies and the recognition of educational methods in workshops that build on practical and applicable clinical scenarios. Among other helpful LOs is to use clinical reasoning methods such as problem-solving in line with the expected objectives in rotations in clinical settings, which was followed only in two schools.

- Expert panel considered medical and educational workload of faculty members to be an obstacle to the implementation problem base learning. It seems that reducing the therapeutic activities of faculties and directing more attention to education should be an essential principle to be considered by planners at the university level.

Systematic review Kilgour et al. showed that clinical students’ viewpoint toward small group, active learning methods were generally positive [22]. Beckman et al.’s research on the use of a collaborative approach to clinical education also showed that while adults prefer collaborative learning and self-learning and that a successful teacher should use facilitated discussions to transfer knowledge and skills, teachers rarely use adult learning principles, such as encouraged interactions, question and answer, and meaningful feedback provision [23]. Using other student-centered methods, such as role-play and question and answer, among others, was another optimal LO practiced only in three schools. Although these interactive educational methods can be among the most straightforward and most accessible educational practices available to teachers, training is organized as a lecture by teachers in most educational clinical settings.

- However, the expert panel considered these practices as feasible, medium-term solutions to improving clinical education. Karbasi et al.’s study on the teaching of how to interact with a patient through role-playing also showed a significant increase in communication skills and care of behaviors in the intervention group [24]. Tayem et al. study showed that the perspectives of medical students toward Role- play demonstrations were positive. They expressed that role- play improved competence in writing a complete prescription [25].

Multimedia and educational films were among other optimal methods, which were used only in two schools. Planners and learners participating in the second sub-study stated that multimedia and educational films are used in limited departments. Together with other simulation methods, these methods can be applied to complement education in cases of inaccessibility or the low number of patients or at the time of risky procedures. However, it seems that utilizing multimedia requires a change in the attitude of teachers. The expert panel considered these practices as education enhancement strategies applicable in the short run. the study Kalani et al. showed that there was no significant difference between the skills gained in the two groups of video training and simulation. However, they showed that the skill level gained by these two groups is significantly different from and higher than the group receiving no intervention (*p* > 0.05) [3].

Patient case Discussion with learners before and after interacting with the patient or performing a surgical procedure was another LO that was considered optimal and applicable by our contributors. Although the participants in the first sub-study stated that they use this educational method, an analysis of the status quo indicated that the tactic is used in only half of the schools (*n* = 4). The teaching of complete patient management is one of the essential requirements of clinical education. It seems that the non-implementation of this educational method is influenced by factors such as the fragmentation of the general wards into sub-specialized ones, the rotation of the learners’ shifts and their presence in a variety of settings in relatively short periods of time, the variability of teachers, and learner rotation based on the wards’ schedule. Even if it is not possible to follow a patient completely, it is suggested that other clinical teaching methods such as small group discussion, virtual methods, and multimedia be used to follow up the patient entirely and to train the whole process of patient management. Managheb et al.’s study comparing role-playing and group discussion on the performance of interns in the transmission of bad news also showed that both group members had an increased mean score in their communication skills. However, comparing the two methods, role-playing proved significantly more effective than the discussion method [26]. Assar et al.’s study on professionalism in education using the demonstration method showed that about 90% of interns assessed the impact of this educational process to be high or very high. Moreover, interns’ performance in the emergency department showed a 68.5% reduction in the inappropriate performance after training as compared with the pre-training period [27].

Expressing experiences for educational purposes is also an optimal LO that was practiced in most faculties. Providing a non-threatening educational environment and building confidence for learners is another beneficial LO that was followed in two-thirds of the schools. Few members of the expert panel in the third sub-study believed that incorrect criteria for the recruitment of faculties and assistants lead to the creation of an inappropriate educational environment in educational settings. Nonetheless, most panel members believed that providing a non-threatening educational environment would be a short-term solution to improving clinical education. Another LO was to encourage learners to participate actively in the clinical services process to learn more, which was followed in one-third of the schools. Some interns participating in the second sub-study considered an intern’s admission in the treatment team and participation in the clinical presentation process as an opportunity to learn and gain experience. Delegating clinical responsibilities to learners in line with their clinical competencies and learning objectives were another beneficial LO, which was performed in one-third of the schools. The findings of this study indicated that learners were not assigned responsibilities based on the educational objectives and the M.D. graduates competencies [28] in most of the schools we examined. The planners are advised to direct attention to reviewing the assignment of responsibilities and having continuous supervision. Shihan et al.’s inquiry into interns’ viewpoint on clinical environment showed that assigning responsibility to interns in the absence of experienced teachers and full-time staff leads to improved learning, ability to make decisions, and an opportunity for clinical empowerment. Despite the empowerment of learners when they are assigned more responsibility in the absence of a senior member of the team, focus group results emphasized the importance of supervision [29]. Kraft et al. also reported that 86% of the students wished to present the findings to the teacher after they had examined the patient alone, and 91% preferred to conduct a clinical examination under the supervision of the trainer [30].

The use of learning tools such as study guide and logbook was another good LO, which was followed in one-third of the schools. Regarding the use of a logbook, most planners and learners believed that this educational tool would not provide an opportunity to learn due to lack of proper supervision by the teacher on how to use the logbook. On the efficacy of internship logbooks, Movafaghi et al.’s study demonstrated that 81% of the teachers and 83% of the students agreed that the accuracy of the information provided in logbooks was less than 60% and that students assessed the accuracy of the logbooks significantly less than the teachers did [31]. On the contrary, Kazemi et al.’s study on the effect of using a study guide on the educational status of clerks and interns showed that the total score of clerks’ and interns’ skills was significantly different in intervention and control groups [32].

Continuously supervision on learners’ performance during clinical tasks was another useful LO, which was performed in two-thirds of schools. However, the learners in the second sub-study agreed that educators rarely have continuous supervision in some wards. On the other hand, members of the expert panel considered continued supervision of learners to assess the attainment of educational objectives a feasible strategy in the short term. Feedback was presented in half of schools. It seems that one of the reasons why the students assessed the teachers’ supervision as not useful was the fact that they did not receive effective feedback. A study by Hem-Stokroos et al. concerning structured clinical experiences showed that all the participants considered the teacher’s supervision of student-patient interaction as a strong stimulus and feedback as a key feature to learning. Nevertheless, they also believed that supervision and feedback were rarely performed by educators [33].

Regarding the administration of the clinical competency assessment (Clinical Internship Completion Test) as an opportunity for learning, our participants from four colleges believed that clinical competency assessment had enhanced learners’ performance. A study conducted on 97 US medical schools between 1993 and 1998 reported that assessment as structured supervision of the ability of clinical learners was performed for only 17 to 23.1% of medical interns. Nevertheless, in a recent study with medical graduates, it was found that only 17 to 39% of clinical examinations conducted by clinical learners were not monitored [34].

### Policy-making to realize LOs

Regarding the policies for LO fulfillment, our participants proposed to define LO as a longitudinal theme in the M.D. curriculum, although this idea was not applied in any of the schools. However, according to the panel members, the adoption of appropriate educational strategies for the design of LOs at schools, based on the M.D. curriculum (approved in 2017) [35], and the specification of LOs in line with the contents of the curriculum were the ways to enhance clinical education, which was feasible in the short run. Moreover, although our participants believed that the vertical integration between basic and clinical sciences provided the ground for creating LOs, it was not implemented in any of the schools and, according to the panel members, it was not feasible. However, the implementation of early clinical encounter programs in some Iranian schools, as an example of the implementation of vertical integration of clinical and basic sciences, has provided satisfaction for learners.

Among other optimal policies for realizing LOs was to launch a record and management system of clinical LOs that is available at all supervisory levels, which did not exist in the schools covered in this study. In addition to providing authorities with the possibility of supervising the educational process, the LOs record system provides the conditions for sharing educational experiences on developing LOs in line with educational and management objectives. However, the panel members believed that it wasn’t feasible. Encouraging educators to effectively manage LOs was another optimal policy that was not observed at the schools. Conducting continuous educational team development on the design of LOs and the continuous assessment of educational team concerning LO management by educational development office of schools were one of the other policies that were practiced in about one-third of the schools. The members of the panel believed that both of these are short-term tactics, the implementation of which on a full scale can be recommended.

One of the limitations of this study in first sub-study was the incorporation of only the experts to learn about the optimum conditions and the lack of access to the opinions of the students and staff. The breadth and diversity of the research community in comparison with the participating sample in all sub-studies was another limitation of this study. In order to reduce impact of this limitation on the results, the participants were selected from all medical schools throughout the country. Shortage of time of the research participants was another limitation in the collection of data as the participants were infused with multiple executive, educational, and treatment responsibilities. We use electronic methods for reducing of this limitation.

## Conclusions

Learners and the clinical LOs provided for them in educational settings are among the main components of learning in clinic settings. Our findings concerning LOs and policies to realize LOs indicated that student-centered clinical learning techniques such as participatory learning, clinical reasoning, and virtual learning methods are optimal tactics that can promote clinical education. Also, LOs such as describing clinical problem on actual patients, discussing the patient case, and expressing teachers’ experiences should be considered besides using interactive and participatory methods. The assignment of responsibility to learners, the direct and indirect learner supervision, the use of learning tools and clinical student assessment were other good LOs and clinical enhancement strategies, which did not enjoy a broad implementation in clinical settings. Also, planning to use learning methods adapted to the objectives in the current clinical settings. Moreover, planning to use LOs in line with the objectives of the M.D. program and clinical settings and its operational management are principles that should be taken into consideration by the planners of the M.D. program.

## Data Availability

The data that support the findings of this study are not publicly available, but can be available from the authors on reasonable request. All questionnaires and other materials are available from the corresponding author on reasonable request.

## Abbreviations

LOs: Learning opportunities
M.D.: Medical Doctor
